# MVDFusion: Multimodal Vehicle Detection in Foggy Weather Using LiDAR and Radar Fusion

**DOI:** 10.3390/s26092663

**Published:** 2026-04-25

**Authors:** Jiake Tian, Yan Gao, Xin Xia, Guoliang Ju, Peijun Ye, Sijie Tang, Hong Wang, Xucong Wang

**Affiliations:** 1 Pengcheng Laboratory, Shenzhen 518055, China; mijiake@mail.scut.edu.cn (J.T.); jugl@pcl.ac.cn (G.J.); yepj@pcl.ac.cn (P.Y.); tangsj@pcl.ac.cn (S.T.); 2 Meteorological Bureau of Shenzhen Municipality, Shenzhen 518040, China; m15818796188@163.com; 3 Shenzhen National Climate Observatory, Meteorological Bureau of Shenzhen Municipality, Shenzhen 518040, China; 4 Shenzhen Key Laboratory of Severe Weather in South China, Shenzhen 518040, China; 5 Shenzhen Key Laboratory of Artificial Intelligence for Meteorological Applications Guangzhou, Shenzhen 518040, China; 6 Guangzhou Institute of Tropical and Marine Meteorology, CMA, Guangzhou 510080, China; hwang@gd121.cn

**Keywords:** millimeter-wave radar, vehicle detection, foggy weather, autonomous driving, sensor fusion

## Abstract

Millimeter-wave (mmWave) radar is widely used for vehicle detection in adverse weather conditions due to its robustness against environmental interference. However, the sparsity of mmWave radar data and the lack of height information significantly limit its broader applicability. To address these challenges, we propose MVDFusion, a multi-modal vehicle detection framework that integrates LiDAR and radar data for robust perception in foggy environments. The proposed framework is designed to fully exploit LiDAR information to compensate for the limitations of sparse radar data. Specifically, two key modules are developed: a radar height query module to enhance height estimation, and a radar–LiDAR query fusion module to improve feature representation. This design enables deep feature-level integration of mmWave radar and LiDAR data. Extensive experiments on the Oxford Radar RobotCar dataset demonstrate that MVDFusion achieves superior performance and robustness under foggy conditions. In particular, it outperforms existing state-of-the-art methods at intersection-over-union thresholds of 0.5, 0.65, and 0.8, achieving detection accuracies of 95.8%, 94.2%, and 81.5%.

## 1. Introduction

The advancement of science, technology, and manufacturing is driving the rapid growth of industrial informatics. In the logistics sector, *Autonomous Vehicles* (AVs) play a pivotal role in this transformation, enhancing the efficiency of freight transportation and reducing traffic accident rates [1]. In real-world applications such as highway transportation, urban logistics, and large-scale warehouse or port operations, AV systems are often required to operate reliably under diverse and challenging environmental conditions, where perception failures may directly lead to safety risks and operational inefficiencies. The *Society of Automotive Engineers* (SAE) classifies autonomous vehicles into several levels, with Level 5 representing full automation, allowing vehicles to operate without human intervention in any environment.

Environmental perception is a critical component of AVs, responsible for detecting and interpreting the dynamic environment surrounding the vehicle to allow accurate and safe decision-making. Cameras and LiDAR are the primary sensors used for environmental perception in AVs [2].

Cameras excel at capturing detailed texture and color information, making them particularly effective for object detection and classification. Camera-based methods have seen notable progress in recent years, as comprehensively reviewed in existing surveys [3,4]. These approaches are generally divided into monocular, stereo, and multi-view categories. In contrast, LiDAR is highly effective in measuring precise distances and reconstructing 3D environments. LiDAR-based methods have advanced significantly in point-based, voxel-based, and projection-based paradigms, with recent overviews provided in the literature [5]. Additionally, researchers have extensively investigated multi-sensor fusion, particularly by combining cameras and LiDAR to leverage their complementary strengths, thereby enhancing the robustness and accuracy of perception systems [6].

However, Cameras and LiDAR are particularly susceptible to challenges posed by adverse weather and lighting conditions, especially in foggy environments [6,7]. Such adverse conditions are frequently encountered in real-world deployments and remain a major bottleneck for the large-scale adoption of AV systems. Fog significantly degrades camera performance by scattering light and reducing image clarity. This degradation can lead to unreliable object detection and missed targets, which may further compromise downstream decision-making processes. For LiDAR, the effective range of performance decreases as fog droplets absorb laser signals from distant objects, further limiting its capability [8]. Moreover, the LiDAR point cloud becomes increasingly noisy due to scattering effects induced by fog [9], resulting in reduced perception accuracy and system robustness in safety-critical scenarios.

In contrast, due to its longer wavelength and wider beam width, *millimeter-wave* (mmWave) radar is less affected by fog. In this paper, we use the term “radar” to refer to “mmWave radar” for consistency. Despite these advantages, radar is constrained by sparse data and the lack of vertical height information due to its horizontal antenna configuration [10]. The height of the radar sensor is typically used to approximate the height values of radar points, which limits its accuracy. Several studies utilizing the ORR dataset have addressed these limitations through LiDAR–radar fusion. For example, TransFusion [11] proposes a two-stage fusion framework for robust 3D object detection in foggy weather, while multi-head attention enhancements of MVDNet [12] and timely fusion approaches [13] further improve feature integration and real-time performance under adverse conditions. However, these methods still suffer from inaccurate height estimation and insufficient handling of radar sparsity, which can lead to localization instability and degraded detection performance in challenging foggy environments. This limitation hinders the effectiveness of existing fusion frameworks and restricts their reliable deployment in adverse weather conditions where precise perception is essential.

Therefore, developing a robust multimodal perception framework capable of reliable operation under adverse weather conditions is essential for the safe deployment and large-scale adoption of autonomous vehicles. To mitigate the limitations of sparse radar data and the absence of height information, we introduce ***MVDFusion***, a multimodal vehicle detection framework for foggy weather that integrates LiDAR and radar data. MVDFusion consists of two main components: ***(1) Radar Height Query Module*** (RHQM), which leverages a Gaussian distance weighting method to evaluate the importance of LiDAR points within a defined query sphere. By incorporating LiDAR data, RHQM reduces radar height estimation errors, enhancing spatial localization accuracy. ***(2) Radar–LiDAR Query Fusion Module*** (RLQFM), which enables effective interaction between radar and LiDAR features. Specifically, the RLQFM alleviates radar sparsity while retaining important local details in radar *Bird’s-Eye View* (BEV) features during convolution. Simultaneously, radar data supplements LiDAR features, enriching the fused representation. Finally, applying a BEV detection head to the integrated features yields more reliable and accurate vehicle detection under foggy conditions.

To evaluate the vehicle detection performance of the MVDFusion framework, we conduct comprehensive experiments on the ORR dataset, which provides high-resolution radar data. Following prior work [8,9], we establish four training and testing scenarios: Mode 1 trains on foggy data and tests on clear data; Mode 2 trains and tests on foggy data; Mode 3 trains on clear data and tests on simulated foggy data; and Mode 4 trains and tests on clear data. The experimental results indicate that MVDFusion outperforms existing methods across several metrics. Mode 1 achieves scores of 95.8%, 94.2%, and 81.5% at *Intersection over Union* (IoU) thresholds of 0.5, 0.65, and 0.8, respectively. Mode 2 reaches scores of 93.2%, 89.6%, and 74.1% at the same thresholds. In Mode 3, MVDFusion shows improvements of 5.5%, 5.1%, and 8% compared to MVDNet [8], while in Mode 4, improvements of 4.3%, 5.3%, and 3.7% are observed.

We conduct comprehensive ablation studies to evaluate the contributions of the RHQM and RLQFM modules. These experiments further investigate the effects of factors such as single-branch input, Gaussian distance weighting, the number of historical frames, and varying fog densities on detection performance. The results demonstrate the robustness of the MVDFusion framework and its ability to achieve superior detection accuracy under diverse challenging conditions.

The main contributions of this paper are:

- We ***propose*** MVDFusion, a multimodal fusion framework designed for vehicle detection in foggy conditions. By leveraging the complementarity of radar and LiDAR, MVDFusion significantly improves detection accuracy in adverse weather conditions.
- We ***design*** the RHQM, which employs Gaussian distance weighting to evaluate the relevance of LiDAR points within a query sphere. By incorporating LiDAR priors, this module corrects radar height estimation errors, resulting in improved spatial localization.
- We ***develop*** the RLQFM to effectively integrate radar and LiDAR features. This module alleviates radar sparsity via LiDAR guidance and enriches LiDAR features with radar attributes, yielding a robust and complementary representation.
- We ***conduct*** extensive experiments on the ORR dataset to evaluate the effectiveness and robustness of MVDFusion, demonstrating its superior detection accuracy under diverse challenging conditions.

The remainder of this paper is organized as follows. Section 2 reviews recent advances in vehicle detection methods categorized by sensor modality. Section 3 presents the proposed MVDFusion framework. Section 4 describes the experimental setup and provides a comprehensive analysis of the results. Finally, Section 5 concludes the paper and discusses future research directions.

## 2. Related Work

### 2.1. Vehicle Detection with Camera Sensor

Camera-based methods are generally categorized into three types: monocular, stereo, and multi-view approaches.

#### 2.1.1. Monocular-Based

Monocular-based methods rely on a single camera image to estimate the 3D position, dimensions, and orientation of objects. Due to the lack of explicit depth information, accurately reconstructing 3D representations remains challenging. Recent works have addressed this limitation through geometry-error priors and decoupled query mechanisms. For instance, MonoDGP employs a Transformer-based framework with decoupled queries and perspective-invariant geometry errors to improve depth estimation [14]. MonoCD leverages complementary depth cues to boost monocular 3D detection accuracy [15]. MonoTAKD further enhances robustness via teaching-assistant knowledge distillation [16]. Overall, these approaches depend primarily on learned priors and sophisticated neural architectures rather than explicit depth sensors.

#### 2.1.2. Stereo-Based

Stereo-based methods utilize image pairs from two cameras with different viewpoints to compute depth information, thereby enabling more accurate 3D object detection and localization. Recent stereo approaches have focused on efficient Transformer architectures and improved cross-view consistency. StereoDETR, for example, introduces a stereo-aware Transformer framework that achieves strong detection performance with computational efficiency [17]. MUSt3R presents a multi-view stereo network that enhances generalization across varying views [18]. CODERS proposes a one-stage framework for category-level object detection, pose estimation, and shape reconstruction from stereo images [19]. While these methods generally provide richer depth cues than monocular approaches, they remain sensitive to lighting changes and adverse weather conditions.

#### 2.1.3. Multi-View-Based

Multi-view methods detect objects across multiple camera views and aggregate features from different perspectives. A common approach involves projecting all 2D views into a unified BEV representation. BEVFormer integrates surround-view camera features with a Transformer-based architecture and has demonstrated excellent performance in BEV-based 3D object detection [20]. CLIP-BEVFormer, which incorporates ground-truth flow and contrastive learning to further improve multi-view BEV detectors [21]. CorrBEV introduces correlation learning with multi-modal prototypes to enhance feature consistency across views [22]. OCBEV proposes an object-centric BEV Transformer for more effective multi-view 3D object detection [23].

Despite these advances in camera-based 3D detection, adverse weather conditions like fog can cause severe light scattering, degraded image quality, and unreliable depth estimation. This fundamental limitation motivates the integration of more robust sensors such as LiDAR and mmWave radar in our proposed MVDFusion framework. To address these challenges, we propose the MVDFusion framework that effectively fuses multi-view camera, LiDAR, and radar data.

### 2.2. Vehicle Detection with LiDAR Sensor

Point cloud feature representation methods can be classified into three main types: point-based, voxel-based, and projection-based. While some studies have explored combining point and voxel features [24,25], this work does not focus on such hybrid approaches.

#### 2.2.1. Point-Based Methods

Point-based methods directly process unordered point cloud data using architectures that emphasize sampling and feature aggregation. These methods extract features from raw point clouds without relying on regular grid structures. For instance, PointRCNN applies binary segmentation to distinguish between foreground and background, followed by bounding box regression with non-maximum suppression to generate high-quality object proposals [26]. Recent advances have focused on more efficient sampling strategies and Transformer-based contextual learning. IA-SSD improves instance-aware sampling for better foreground–background separation [24]. PointTransformer enhances set-structured data processing with self-attention mechanisms [27]. PTv2 introduces an improved point Transformer with better local feature aggregation and scalability [28].

#### 2.2.2. Voxel-Based Methods

Voxel-based methods discretize point cloud data into a regular 3D grid structure, analogous to pixels in a 2D image. PointPillars organizes point clouds into vertical columns (pillars) along the x- and y-axes [29], converting them into 2D pseudo-images for simplified feature learning. SECOND incorporates sparse convolutional layers to reduce computational overhead while improving processing speed [30]. Recent works have extended these foundations with more efficient sparse convolutions and attention mechanisms. Voxel Transformer further enhances efficiency by integrating attention and fast query algorithms [31]. 3DPillars proposes a two-stage pillar-based framework to better preserve 3D structures while maintaining real-time performance [32]. SPVCNN introduces a sparse point-voxel convolution network that achieves a better balance between accuracy and efficiency [33].

#### 2.2.3. Projection-Based Methods

Projection-based methods project point cloud data onto specific views and apply image-based techniques for detection. PIXOR specializes in real-time 3D object detection by combining 3D occupancy tensors with LiDAR reflectance data [34], using deep neural networks for efficient object localization and classification. Recent projection-based approaches have explored multi-view consistency and BEV refinements for better accuracy in complex scenes. BEVDepth integrates depth estimation into BEV representation to improve projection-based detection [35]. BEVFormer enhances multi-view projection with spatiotemporal Transformers for more robust feature aggregation [20]. LiDAR-BEVMTN presents a real-time multi-task network for joint detection and segmentation in BEV representation [36].

### 2.3. Vehicle Detection with Multimodal Sensors

Multimodal sensors enhance vehicle detection by providing complementary and redundant information, leading to improved performance. While most existing fusion approaches focus on LiDAR and cameras, such as BEVFusion [37], DeepFusion [38], SimpleBEV [39], and MV2DFusion [40], their effectiveness is significantly limited under extreme weather conditions. In contrast, radar remains largely unaffected by fog due to its longer wavelength, making LiDAR–radar fusion a promising direction for robust perception in adverse weather.

Early efforts include RadarNet [10], which integrates sparse radar points with LiDAR using CNNs for 360° detection, LiRaNet [41] for trajectory prediction, and DEF [7] for early fusion of LiDAR, camera, and radar. However, these methods often suffer from radar’s low resolution and sparsity.

The ORR dataset, with its high-resolution rotating mmWave radar and synchronized LiDAR, has served as a key benchmark for foggy conditions. Representative works on ORR include MVDNet [8] (attention-based lightweight fusion for foggy detection), Bi-LRFusion [42] (bidirectional query-based fusion to mitigate sparsity and height issues), and ST-MVDNet [9] (mutual learning for sensor-agnostic robustness).

More recent studies on the ORR dataset have further advanced LiDAR–radar fusion for foggy vehicle detection. For instance, TransFusion [11] proposes a two-stage robust fusion framework. Multi-head attention enhancements of MVDNet [12] improve feature integration under adverse conditions, while timely fusion approaches [13] address the temporal misalignment between radar and LiDAR. Nevertheless, traditional high-resolution mmWave radar (as used in ORR) remains widely deployed in practice due to cost and hardware maturity. Two persistent challenges in fog-oriented fusion with such radar are: (1) the lack of reliable height information, leading to localization instability; (2) severe sparsity, which hinders robust feature representation.

To explicitly address these challenges on the ORR dataset—particularly the limitations summarized in Table 1—we propose MVDFusion. Unlike approaches that rely on approximate height or general cross-attention, our framework introduces a Radar Height Query Module with Gaussian-weighted LiDAR guidance for physically grounded height reconstruction, and a Radar–LiDAR Query Fusion Module with bidirectional sparsity-aware interaction. This design enables more effective feature-level integration tailored to traditional mmWave radar in foggy conditions, ultimately feeding fused features into a BEV detection head for accurate vehicle detection.

## 3. Methodology

**Figure 1:**
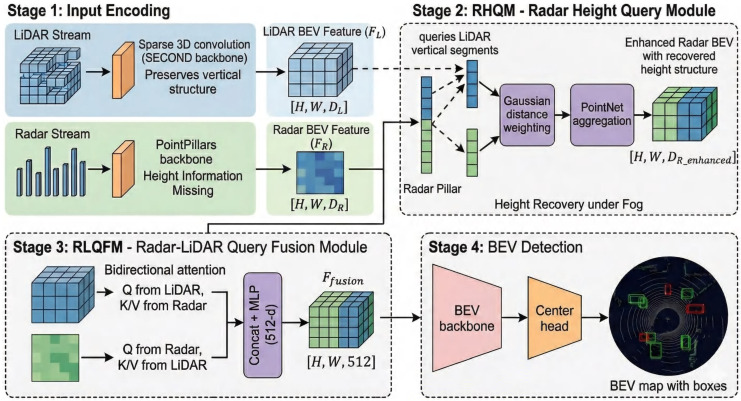
The system’s overall workflow consists of several key components: input encoding, the radar height query module, the radar–LiDAR query fusion module, and the BEV detection head.

Figure 1 illustrates the overall workflow of MVDFusion, which incorporates two novel query-based fusion modules as its core innovation.

Unlike existing query-based or attention-based fusion methods that mainly focus on generic cross-modal feature interaction, our design is specifically motivated by two critical challenges in radar–LiDAR fusion under adverse weather conditions: (1) the lack of reliable height information from radar, and (2) the severe sparsity and uneven spatial distribution of radar measurements. To address these issues, we propose a joint design consisting of the Radar Height Query Module (RHQM) and the Radar–LiDAR Query Fusion Module (RLQFM). The RHQM explicitly reconstructs radar height using LiDAR-guided Gaussian-weighted queries, while the RLQFM performs sparsity-aware bidirectional feature interaction between the two sensors. This problem-driven design distinguishes our approach from generic cross-attention-based fusion frameworks.

Section 3.2 describes the radar height query module, focusing on the correction of radar height data using precise 3D LiDAR information and the application of a Gaussian weighting model to prioritize points within the query sphere. Section 3.3 presents the radar–LiDAR query fusion module, which leverages a bidirectional query mechanism to fully exploit the complementary strengths of both sensors. Additionally, Section 3.1 provides an overview of the encoders for the radar and LiDAR branches, and Section 3.4 discusses the BEV detection head.

### 3.1. Input Encoder

#### 3.1.1. LiDAR Encoder

Consistent with the approach used in SECOND [30], we partition LiDAR points into voxels and encode the point features within each grid using a *Multi-Layer Perceptron* (MLP). Each grid, known as a voxel, represents a discrete 3D unit, and the features encoded within the voxel are referred to as voxel features. These voxel features are processed by a 3D voxel backbone network that incorporates sparse and submanifold convolutions to enable efficient feature extraction. To generate the BEV feature map, voxel features from non-empty grids are aggregated along the z-axis (height), resulting in a 2D feature map represented as $F_{L}\in R^{C_{L}\times H\times W}$, where *H* and *W* denote the spatial dimensions of the BEV, and $C_{L}$ indicates the number of LiDAR channels.

#### 3.1.2. Radar Encoder

Radar data in the ORR dataset is captured as intensity images with lossless compression, lacking additional feature attributes such as object radial velocity, dynamic properties, clustering validity, and false alarm probabilities. In accordance with [42], radar images are converted into point cloud data, which includes 3D coordinates (x,y,z), *Radar Cross-Section* (RCS), and timestamps (*t*), using a feature extractor. The z-axis values of radar points are set to 0 by default. For encoding radar features, we adopt the PointPillars method [29], which transforms radar data into pseudo-images within the BEV space. A pillar feature network is then employed to extract radar features, producing the radar BEV feature map $F_{R}\in R^{C_{R}\times H\times W}$, where $C_{R}$ represents the number of radar channels.

### 3.2. Radar Height Query Module

**Figure 2:**
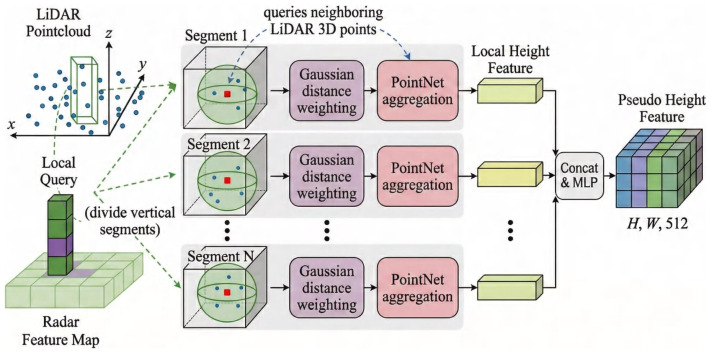
The radar height query module utilizes a spherical query mechanism combined with Gaussian distance weighting to accurately reconstruct radar height information.

As depicted in Figure 2, we address the challenge of missing radar height data, as mentioned in Section 1, by proposing the Radar Height Query Module (RHQM) to explicitly reconstruct the missing vertical information in radar data.

Unlike existing methods that either ignore radar height or rely on coarse approximations, RHQM explicitly reconstructs radar height using LiDAR-guided local queries, providing a physically grounded and locally adaptive height estimation mechanism. This method not only improves height accuracy but also ensures better integration of radar and LiDAR data in challenging environments, such as fog. This design effectively introduces a localized geometric prior that bridges sparse radar observations with dense LiDAR structure.

The process proceeds as follows: First, for each non-empty radar BEV grid, the vertical column (pillar) is divided along the z-axis into multiple cubic segments, each having its center as a query point. Assuming the radar BEV feature map grid size is r×r, and taking the *i*-th non-empty grid as an example, we define the spherical query radius as r/2 to prevent redundancy and computational inefficiency from querying the same LiDAR points multiple times. The number of segments, *M*, is calculated as the ratio of the column height *h* to the grid size *r*, i.e., M=h/r. Consequently, the height of the query point in the *j*-th segment is given by the formula

(1) $$h_{qi,j}=h_{Lmin}+2r\left(j-1\right).$$

Regarding the query radius, this value is set based on the BEV grid size, specifically half of the grid resolution. This design is commonly adopted in BEV-based methods, ensuring spatial locality while avoiding excessive overlap.

During the spherical query process, LiDAR points scattered within the sphere exhibit varying distributions, and not all points contribute equally to the height information. To address this, we propose a Gaussian distance weighting method to differentiate the importance of LiDAR points near the query point. The core idea is that points closer to the sphere’s center are deemed more significant, and thus assigned higher weights. This Gaussian weighting mechanism has been empirically optimized in our experiments, and we observe that the method remains stable across a reasonable range of σ values. Small variations in σ do not lead to significant performance degradation, ensuring the robustness of the method.

For any point $x_{i}$ within the sphere, its weight $w_{i}$ is determined by the following Gaussian function, subject to a distance constraint

(2) $$w_{i}=\left\{\begin{array}{cc} \exp\left(-\frac{∥x_{i}{-c∥}^{2}}{2\sigma^{2}}\right), & \mathrm{if}\;∥x_{i}-c∥\;\leq r\\ 0, & \mathrm{otherwise}. \end{array}\right.$$

where $∥x_{i}-c∥$ denotes the Euclidean distance between the point $x_{i}$ and the sphere center *c*. The standard deviation σ of the Gaussian distribution controls the rate at which the weight decreases with increasing distance. We empirically observe that the method remains stable across a reasonable range of σ values, indicating robustness to parameter variations.

Through spherical queries, LiDAR points within each designated spherical region are grouped. For each segment, we use the PointNet model to aggregate local height features from the grouped LiDAR points. The computation is given as follows

(3) $$F_{s}=\max_{k=1,2,\ldots ,K}\left\{\mathrm{MLP}\left(n_{s}^{k}\right)\right\},$$

Finally, the height features from all segments are aggregated and passed through an additional MLP to generate features that match the dimensions of the radar BEV feature map

(4) $${\tilde{F}}_{H}^{l}=\mathrm{MLP}\left(\mathrm{Concat}\left({\left\{F_{s}\right\}}_{s=1}^{M}\right)\right).$$

Through this explicit height reconstruction process, RHQM effectively transforms radar data from a 2.5D representation into a more complete 3D-aware feature space, improving spatial consistency and localization accuracy.

### 3.3. Radar–LiDAR Query Fusion Module

LiDAR features $F_{L}\in R^{C_{L}\times H\times W}$ provide rich spatial information, whereas radar features $F_{R}\in R^{C_{R}\times H\times W}$ are relatively sparse. This inherent imbalance between dense LiDAR features and sparse radar features poses a major challenge for existing fusion methods.

Unlike standard cross-attention mechanisms that treat different modalities in a symmetric manner, the proposed RLQFM explicitly accounts for this imbalance through a sparsity-aware bidirectional query strategy. In particular, LiDAR features guide the completion of sparse radar regions, while radar features provide complementary signals to refine LiDAR representations. This asymmetric design fundamentally differs from conventional symmetric fusion frameworks.

**Figure 3:**
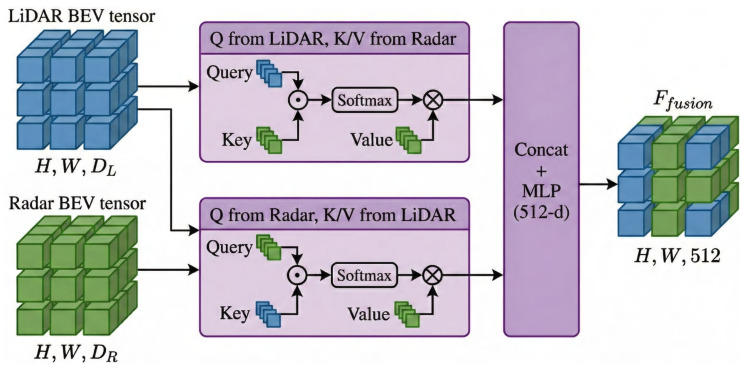
The radar–LiDAR query fusion module facilitates information exchange between the two modalities using a bidirectional query approach and finally concatenates the two modality branches to achieve fusion.

To enable effective interaction between radar and LiDAR features, we propose a query-based mechanism, as depicted in Figure 3. The LiDAR feature map $F_{L}$ queries the non-empty spatial positions in the radar feature map $F_{R}$, thereby facilitating the transfer of radar-specific information. Conversely, the radar feature map queries the corresponding regions in $F_{L}$, enabling dense-to-sparse information completion. This bidirectional querying mechanism enables distribution-aware feature interaction, allowing both sensors to effectively exploit complementary information while explicitly addressing radar sparsity.

The Query (Q), Key (K), and Value (V) matrices are generated through learnable linear transformations. The attention mechanism computes the similarity between queries and keys using a scaled dot-product operation, followed by a softmax function to normalize the attention weights

(5) $$A_{L\to R}=\mathrm{softmax}\left(\frac{Q_{L}K_{R}^{T}}{\sqrt{d_{k}}}\right),$$

(6) $$A_{R\to L}=\mathrm{softmax}\left(\frac{Q_{R}K_{L}^{T}}{\sqrt{d_{k}}}\right),$$

where $d_{k}$ is the dimensionality of the key vector. These attention weights *A* are applied to the value matrices *V*, yielding updated feature representations ${\mathrm{Output}}_{R}$ and ${\mathrm{Output}}_{L}$, which encapsulate complementary spatial and semantic information from both modalities. The L→R path emphasizes dense-to-sparse feature completion, while the R→L path injects radar-specific complementary cues into the LiDAR representation.

Subsequently, the updated radar and LiDAR features are concatenated along the channel dimension and processed through an MLP with a ReLU activation function. The MLP reduces the high-dimensional concatenated features to a fixed 512-dimensional vector for efficient downstream processing

(7) $$F_{\mathrm{fusion}}=\mathrm{MLP}\left(\mathrm{Concat}\left({\mathrm{Output}}_{R},{\mathrm{Output}}_{L}\right)\right).$$

The MLP consists of two fully connected layers, with the first layer employing batch normalization and ReLU activation to ensure stable feature scaling and nonlinearity. Dropout is applied after the first layer to mitigate overfitting and improve generalization. The second layer outputs the final fused features $F_{\mathrm{fusion}}$, which integrate complementary spatial and semantic details from both modalities. By explicitly modeling the imbalance between dense and sparse modalities, RLQFM achieves more effective feature fusion than conventional attention-based methods, leading to improved robustness and detection accuracy under adverse weather conditions.

### 3.4. BEV Detection Head

Finally, the fused features are fed into a BEV detection network to produce detection results. The network consists of a BEV feature extraction backbone and a detection head. The backbone uses multiple 2D convolutional blocks to extract features, which are then passed to the detection head. We adopt a category-specific center heatmap head to predict object centers, along with regression heads for size, orientation, and velocity. The overall loss is jointly optimized following CenterPoint [43].

## 4. Experimental Results and Analysis

### 4.1. Dataset and Experimental Setup

#### 4.1.1. Dataset

We utilize the Oxford Radar RobotCar (ORR) dataset [44], a radar extension of the well-established Oxford RobotCar Dataset developed by the Oxford Robotics Institute, covering diverse weather, traffic, lighting, and seasonal conditions (including rain, snow, and overcast days that facilitate fog simulation). This makes the dataset particularly suitable for studying multimodal perception under adverse weather conditions.

It contains 8862 synchronized LiDAR–radar frame pairs (7071 for training and 1791 for testing, with no geographical overlap), featuring:

- Dual Velodyne HDL-32E 3D LiDARs (32 beams each, 0.33° angular resolution, 10 Hz scanning rate);
- Navtech CTS350-X millimetre-wave FMCW rotating radar (360° field-of-view, 0.9° angular resolution, 4.38 cm range resolution, up to 163 m range, 4 Hz scanning rate, lossless PNG intensity images);
- Additional sensors (cameras, GPS/INS) and optimized ground-truth radar odometry.

Unlike many automotive radar datasets that rely on low-resolution or forward-facing radar sensors, ORR employs a high-resolution rotating radar with full 360° coverage, enabling more reliable evaluation of radar sparsity and height estimation issues. Compared with other publicly available datasets, ORR is particularly well-suited for our problem setting. For instance, nuScenes includes 77 GHz automotive radar but with much lower resolution and limited fog simulation; KITTI and Waymo lack radar data entirely; RADIATE and Boreas provide radar measurements but primarily focus on SLAM or place recognition tasks with different sensing setups and evaluation protocols. This is because it uniquely satisfies three key requirements of our study: (1) high-resolution rotating radar for analyzing sparsity and feature representation, (2) synchronized high-resolution LiDAR for accurate height reconstruction, and (3) real-world urban scenarios with diverse weather conditions for evaluating robustness under fog.

To the best of our knowledge, ORR is the only publicly available dataset that simultaneously fulfills all these requirements, making it the most appropriate benchmark for evaluating the proposed MVDFusion framework. Moreover, it serves as the standard benchmark for prior fog-oriented fusion works (e.g., MVDNet [8], Bi-LRFusion [42], ST-MVDNet [9]), ensuring fair and direct comparisons.

#### 4.1.2. Data Processing

The ORR dataset contains extensive unlabeled raw data [8]. The radar performs a 360° scan every 0.25 s with 0.9° angular resolution, while the dual LiDARs scan at 0.05 s intervals with finer angular resolution. These temporal and resolution differences pose synchronization challenges.

Following prior studies [8,9,42], we employ a SLAM-based alignment method to correct misalignments by estimating relative pose between radar and LiDAR points. Additionally, we aggregate five consecutive LiDAR frames into one unified frame using a sector scan approach [8] to match the radar timestamp, ensuring temporal synchronization.

To improve model generalization, we apply point cloud data augmentation including random rotation, translation, and scaling. We remove LiDAR points beyond a distance threshold with 50% probability on training samples to mimic fog-induced range reduction and noise. For foggy condition simulation, we adopt the fog model from DEF [7], which removes LiDAR points beyond a distance threshold with 50% probability during training to mimic fog-induced range reduction and noise.

For evaluation, we adopt mean Average Precision (mAP) from the COCO framework [8], reporting results at IoU thresholds of 0.5, 0.65, and 0.8. We note that more comprehensive metrics such as the nuScenes Detection Score (NDS), Average Translation Error (ATE), and Average Scale Error (ASE) are commonly used in datasets like nuScenes; however, these metrics require specific annotations and evaluation protocols that are not available in the ORR dataset. Following prior works on ORR (e.g., MVDNet, Bi-LRFusion, and ST-MVDNet), we adopt mAP as the primary evaluation metric to ensure fair and consistent comparison.

#### 4.1.3. Experimental Setup

We perform our experiments on a high-performance computing setup featuring 8 × A100 GPUs and implement our model in PyTorch. The model is trained for 40 epochs with a batch size of 2, using an SGD optimizer initialized with a learning rate of 0.01, which decreases by a factor of 0.1 every 20 epochs. For LiDAR data, we define the spatial range as [−69.12, 69.12] meters along the X and Y axes and [−5.0, 2.0] meters along the Z axis, with a voxel size of [0.32, 0.32, 7.0] meters. Radar data, initially provided in image format, is converted into point clouds using the feature extraction method described in [45]. Each point includes attributes such as x, y, z coordinates, RCS derived from the image’s grayscale value, and timestamps, with the z-coordinate fixed at zero. To address noise and artifacts from multipath effects, we employ a geometric probability filter [42], reducing the radar point count to around 1000 points per frame.

### 4.2. Baseline Comparison

To rigorously evaluate the vehicle detection capability of MVDFusion, extensive experiments were conducted under four training/testing settings and at IoU thresholds of 0.5, 0.65, and 0.8. Specifically, Mode 1 involves training under foggy conditions and testing in clear weather; Mode 2 involves both training and testing under foggy conditions; Mode 3 uses clear-weather training and foggy-weather testing; and Mode 4 involves both training and testing under clear conditions.

**Figure 4:**
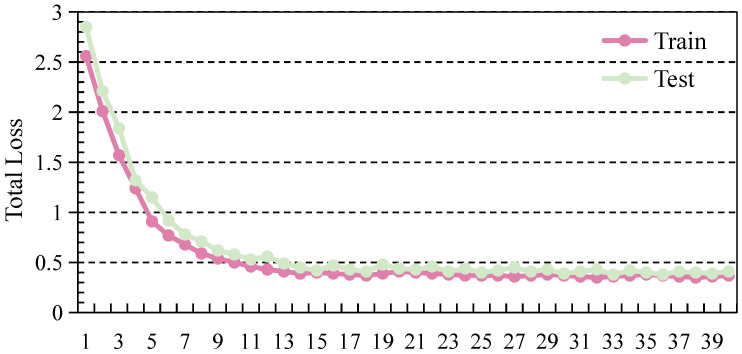
Training and testing loss curves over 40 sampled epochs, demonstrating the stable convergence and strong generalization capability of the proposed model.

The experimental results, as shown in Table 2, demonstrate that methods utilizing both LiDAR and radar (L + R) consistently outperform those relying solely on LiDAR (L), particularly under challenging conditions such as foggy weather. This observation indicates that radar provides complementary and robust range information that remains reliable under degraded visibility, thereby compensating for the limitations of LiDAR in adverse weather. The MVDFusion model achieves state-of-the-art performance across different training/testing settings and IoU thresholds. Specifically, when trained with foggy-weather data and tested in clear weather, MVDFusion achieves 95.8%, 94.2%, and 81.5% at IoU thresholds of 0.5, 0.65, and 0.8, respectively. Under foggy testing conditions, the corresponding performance reaches 93.2%, 89.6%, and 74.1%. These consistent improvements across different settings suggest that MVDFusion learns more generalizable feature representations and maintains strong robustness under domain shifts between clear and foggy environments. Figure 4 shows the training and testing loss curves over 40 epochs. The curves demonstrate stable convergence without significant overfitting, indicating good generalization ability of MVDFusion.

A higher IoU threshold of 0.8 poses a greater challenge for all methods. While PointPillars achieves relatively strong localization performance at higher IoU thresholds, it still outperforms some radar–LiDAR fusion methods such as DEF [7]. This result can be attributed to the fact that DEF is primarily optimized for front-view data, which limits its performance in more complex 360° scenarios. Notably, the advantage of MVDFusion becomes more pronounced at higher IoU thresholds, indicating its superior localization accuracy. In contrast, MVDFusion shows substantial improvements over recent methods, with mAP gains of 6.9% and 5.2% at IoU 0.8 for modes 1 and 2, respectively, compared to Bi-LRFusion and ST-MVDNet. This improvement is mainly attributed to the explicit radar height modeling in RHQM, which enhances geometric consistency, and the bidirectional feature interaction in RLQFM, which alleviates radar sparsity.

Recent fusion approaches on the ORR dataset, such as TransFusion [11], multi-head attention enhanced MVDNet [12], and Timely Fusion [13], have made progress in foggy conditions, yet they still face limitations in precise height estimation and sparsity handling. In comparison, MVDFusion consistently demonstrates stronger robustness and reliability whether the testing condition is clear or foggy.

To further evaluate model robustness under extreme weather conditions, we conduct experiments using training data that contain only clear-weather samples, i.e., LiDAR data without fog-induced noise. The results in Table 2 reveal a general performance drop for both single-modality and radar–LiDAR fusion models when foggy conditions are not included during training. This performance degradation highlights the importance of training data diversity and the necessity of modeling adverse conditions during training. For example, PointPillars achieves an mAP of 85.8% at IoU 0.5 under clear-weather training, which decreases to 71.3% under foggy testing. Similarly, MVDNet achieves 90.9% at IoU 0.5 when trained under foggy conditions, but only 87.2% when trained and tested under clear-weather settings. These results indicate that models trained only on clear-weather data have limited generalization ability under degraded visibility, as they fail to capture the distribution shift introduced by fog.

In contrast, MVDFusion demonstrates stronger robustness, achieving 92.7% in clear-weather testing and 82.3% in foggy-weather testing at IoU 0.5 under the clear-only training setting. This further validates that the proposed fusion strategy effectively exploits complementary sensor information to improve robustness across varying environmental conditions. These results indicate that incorporating fog-like degradation during training significantly improves robustness, especially for detection tasks in adverse weather.

**Figure 5:**
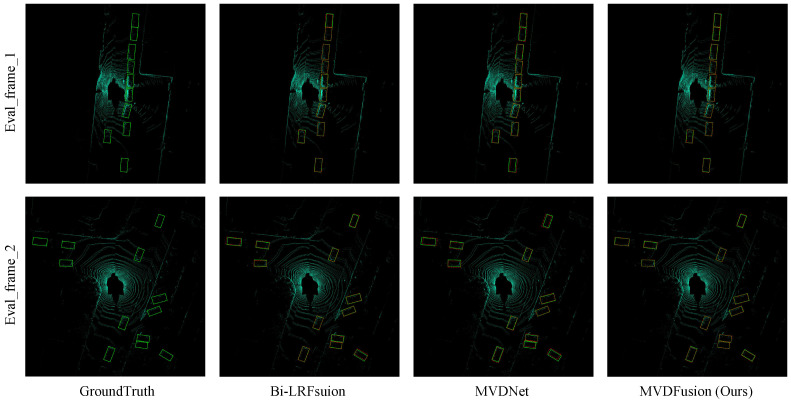
Qualitative comparison of detection results under foggy conditions. From left to right are GroundTruth, Bi-LRFusion, MVDNet, and the proposed MVDFusion. MVDFusion produces more accurate bounding boxes and fewer missed detections, especially for distant and partially occluded vehicles.

Figure 5 compares the detection outputs of Bi-LRFusion, MVDNet, and MVDFusion against the ground truth under foggy conditions. As shown in Figure 5, existing methods such as Bi-LRFusion and MVDNet tend to miss distant objects or produce less accurate bounding boxes, which is likely caused by radar sparsity and unreliable height information. In contrast, MVDFusion achieves more accurate localization and more complete detections, particularly for small or partially occluded vehicles. This observation is consistent with the design of the proposed method: RHQM improves radar height estimation, while RLQFM enhances feature representation through cross-modal interaction. These qualitative observations are also consistent with the quantitative improvements reported in Table 2.

**Figure 6:**
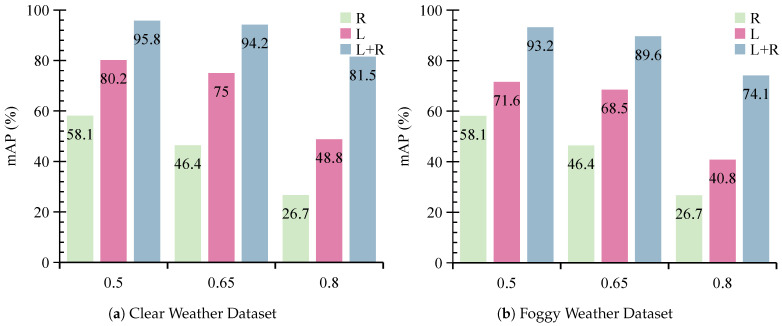
The detection performance of various modality branches in MVDFusion under the foggy conditions: (**a**) represents the test set with clear weather data, and (**b**) represents the test set with foggy weather data.

**Figure 7:**
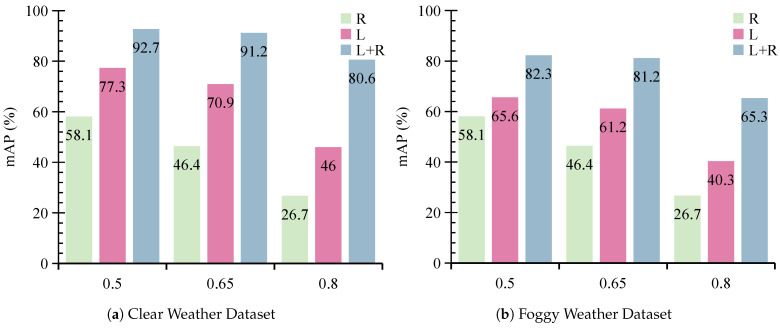
The detection performance of various modality branches in MVDFusion under clear conditions: (**a**) represents the test set with clear weather data, (**b**) represents the test set with foggy weather data.

We further evaluate the performance of MVDFusion by comparing it with its individual modality branches. As illustrated in Figure 6 and Figure 7, the Radar-Only branch exhibits suboptimal performance across all evaluation metrics. Specifically, it achieves an mAP of only 58.1% at IoU 0.5, 46.4% at IoU 0.65, and 26.7% at IoU 0.8. This highlights that radar alone lacks sufficient spatial structure and reliable height information for accurate object localization. In contrast, the LiDAR-Only branch demonstrates much stronger performance than the Radar-Only branch. However, when trained using foggy data, the LiDAR-Only branch shows performance drops of 9.6%, 6.5%, and 8.0% at IoU thresholds of 0.5, 0.65, and 0.8, respectively, when evaluated under clear-weather conditions. Similarly, when trained under clear-weather conditions, its performance drops by 11.7%, 9.7%, and 5.7% under foggy testing at the same IoU thresholds. These findings demonstrate that LiDAR is highly sensitive to environmental conditions and its performance degrades significantly in fog due to signal attenuation and increased noise. These results further confirm that, although LiDAR performs well under clear conditions, its effectiveness is substantially reduced in adverse weather such as fog.

### 4.3. Ablation Study

We conduct extensive ablation experiments on the MVDFusion framework to assess the individual contributions of its essential components, including the Data Augmentation Module (DAM), the RHQM, and the RLQFM. Moreover, we evaluate the impact of incorporating historical frames, different levels of fog density, and the Gaussian weighting strategy for height completion.

#### 4.3.1. Module Contributions

We perform an in-depth ablation study to evaluate the contributions of the DAM, the RHQM, and the RLQFM to the overall performance of MVDFusion. As shown in Table 3, all three modules contribute to the final performance improvement. Starting from the baseline model without any of these modules, the detection accuracy is relatively limited, especially at higher IoU thresholds such as 0.8. As the modules are progressively introduced, the performance consistently improves across all settings, demonstrating the effectiveness of each component.

The DAM employs rotation-based data augmentation techniques, which help the model learn more robust and generalized features. As shown in Table 3, introducing DAM leads to consistent gains across different IoU thresholds. For example, under the Clear + Foggy setting at IoU 0.5, the performance improves from 87.3% to 89.7%, indicating that data augmentation enhances generalization under both clear and adverse weather conditions.

The RHQM improves spatial localization by using LiDAR point cloud data to adjust the height information of radar inputs, leading to a clear improvement in localization accuracy. In particular, after introducing RHQM, the improvements become more pronounced at higher IoU thresholds, indicating that accurate height modeling is especially important for precise bounding box estimation.

The RLQFM is especially important for detection in foggy conditions. Using its query-based approach, this module compensates for spatial inaccuracies in detection and integrates complementary radar and LiDAR features via a bidirectional fusion strategy. As shown in Table 3, incorporating RLQFM yields the most significant performance gains, particularly in foggy conditions and at higher IoU thresholds. This demonstrates that the proposed bidirectional fusion strategy effectively alleviates radar sparsity and enhances feature representation in challenging environments.

#### 4.3.2. Impact of Historical Data Frames

Multi-frame data provides temporal correlation information, enabling the model to capture dynamic object variations and thereby improving object recognition accuracy. However, excessively long temporal windows may introduce substantial redundant information, and outdated positional or state information can interfere with the detection results of the current frame, ultimately reducing the model’s accuracy. Based on these considerations, we adjust the number of LiDAR and radar frames to investigate the impact of historical data on the performance of MVDFusion.

**Figure 8:**
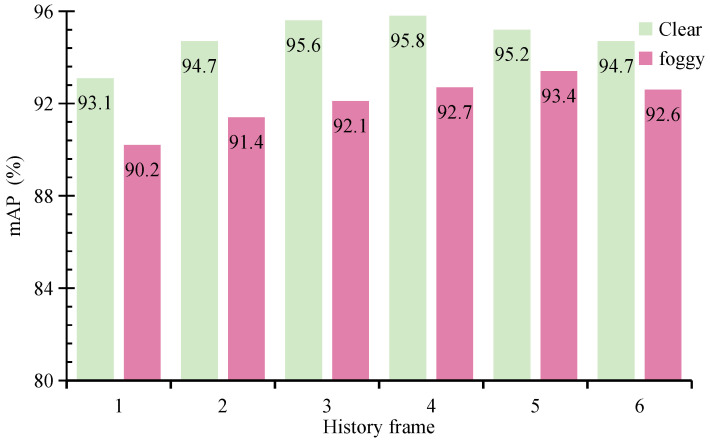
The impact of historical frames on the performance of MVDFusion under the foggy training set and clear test set.

As shown in Figure 8, the performance of MVDFusion on both the clear weather and foggy weather test sets improves progressively as the number of historical frames increases from 1 to 4. However, when the number of frames reaches 5 or 6, a noticeable decline in performance is observed. This decline is attributed to redundant or outdated information interfering with the representation of the current frame. After a comprehensive analysis of the experimental results, we select a history frame count of 4, striking a balance between temporal correlation and information redundancy, thereby maximizing the detection performance.

#### 4.3.3. Effect of Fog Density

The influence of fog density is significant. Fog droplets absorb and scatter reflected signals from distant targets, thereby reducing the detection range of LiDAR systems. For instance, dense fog with a density of 0.08· m^−1^ limits the visible range of the Velodyne HDL32-E LiDAR to just 15 m [8]. It is worth noting that increasing fog density not only reduces the effective sensing range but introduces severe data sparsity and incomplete spatial observations in LiDAR measurements. Therefore, this experiment can be interpreted as a controlled evaluation of the model under varying levels of sensing range degradation and sparsity, which directly relates to the core challenges addressed in this work.

**Figure 9:**
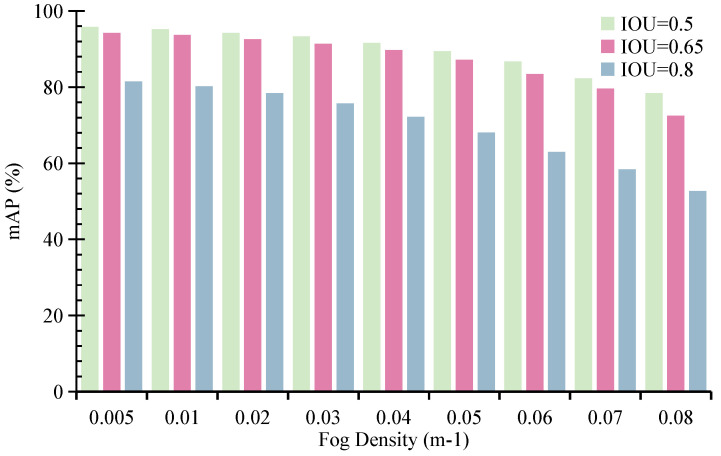
The impact of fog density on the performance of MVDFusion under the foggy training set and clear test set.

We examine how varying fog densities, from 0.005· m^−1^ to 0.08· m^−1^, affect the performance of the MVDFusion detector. As illustrated in Figure 9, when the detector is trained on foggy data and tested on clear data, its performance under IoU thresholds of 0.5, 0.65, and 0.8 declines notably as fog density increases. At a fog density of 0.08· m^−1^, the mAP falls below 0.6. Despite this degradation, MVDFusion consistently achieves the highest performance across all fog densities, indicating that the proposed method is robust to both reduced sensing range and increased sparsity.

In comparison to the results reported in MVDNet, MVDFusion achieves the highest mAP of 78.2% at the maximum fog density of 0.08· m^−1^ under the IoU 0.5 condition, exhibiting the smallest decline in detection accuracy. This robustness can be attributed to the explicit height reconstruction in RHQM, which compensates for missing geometric information, and the sparsity-aware fusion in RLQFM, which effectively integrates complementary information from LiDAR and radar under degraded sensing conditions. This result further demonstrates the robustness of MVDFusion in detecting targets under extreme weather conditions.

#### 4.3.4. Effect of Gaussian Weighting

**Figure 10:**
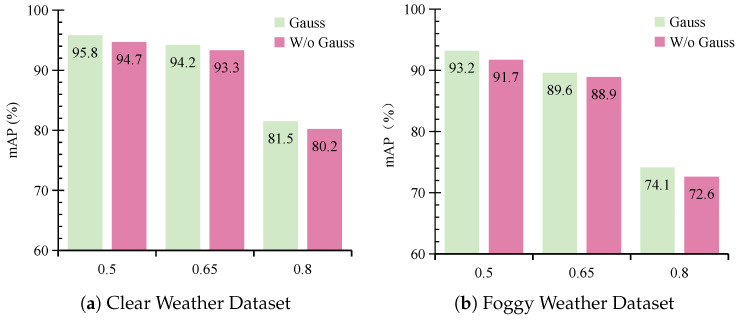
The impact of Gaussian weighting on the performance of MVDFusion under foggy training conditions. (**a**) represents the test set with clear weather data, and (**b**) represents the test set with foggy weather data. “W/o” denotes the variant without Gaussian weighting.

In the RHQM, we introduce a Gaussian weighting strategy based on the distances to the query point in order to prioritize the significance of points within the query region. By assigning Gaussian weights that decrease with distance, this approach ensures that points closer to the query point contribute more substantially to height completion, thereby enhancing the precision of radar height correction. Experiments conducted under IoU thresholds of 0.5, 0.65, and 0.8 demonstrate the effectiveness of this method. Results for data Mode 1 (foggy training and clear testing) are illustrated in Figure 10a, and results for data Mode 2 (foggy training and foggy testing) are illustrated in Figure 10b.

The findings reveal that the Gaussian weighting method outperforms the equal-weight strategy, in which all points are assigned identical weights. This indicates that assigning higher importance to points closer to the query center leads to more accurate height estimation. As a result, the spatial consistency of the fused features is improved, which further enhances the overall detection performance.

## 5. Conclusions

In this paper, we propose MVDFusion, a robust multimodal fusion framework for vehicle detection under foggy conditions. The key innovation of MVDFusion lies in the development of two core modules: ***the radar height query module*** and ***the radar–LiDAR query fusion module***. These modules explicitly leverage the complementary strengths of radar and LiDAR to enable effective feature-level fusion of radar intensity maps and LiDAR point clouds. As a result, the proposed framework effectively addresses the lack of height information and the sparsity of radar measurements, leading to consistently improved detection performance on the ORR dataset. From the experimental results, we draw the following key findings: (1) integrating radar with LiDAR significantly improves robustness under adverse weather compared to LiDAR-only methods; (2) accurate radar height modeling is essential for reliable localization, particularly at higher IoU thresholds; (3) addressing radar sparsity through feature-level fusion further enhances detection performance in complex environments. These findings highlight that explicitly modeling radar geometry and designing sparsity-aware fusion mechanisms are critical for building reliable perception systems in real-world autonomous driving in adverse weather.

In future work, we plan to further investigate the following directions to enhance the performance and applicability of the proposed MVDFusion framework:

- **Expanding to Diverse Weather Conditions:** While the current model focuses on foggy conditions, we aim to extend its applicability to other adverse weather scenarios such as rain, snow, and sandstorms. This will involve expanding the training data and designing more robust augmentation strategies to improve generalization across diverse conditions.
- **Handling Sensor Failures:** In real-world scenarios, sensors may malfunction due to hardware issues or environmental interference. We plan to develop fault-tolerant mechanisms by leveraging multi-sensor redundancy, enabling reliable perception even when certain sensors are partially degraded or unavailable.
- **Developing More Effective Fusion Strategies:** Although current multimodal fusion methods have shown promising results, there remains room for improvement. We plan to explore more adaptive fusion strategies, such as dynamic cross-modal interaction and task-aware fusion mechanisms, to further enhance feature representation and integration efficiency.
- **Extensive Validation Across Diverse Conditions:** To further evaluate generalizability, we plan to validate the proposed method on additional datasets and under more diverse scenarios, including varying target distances, traffic densities, and radar sparsity levels. In particular, we will extend our evaluation to 4D radar datasets that provide richer information such as Doppler velocity and elevation, in order to further assess the effectiveness and adaptability of the proposed framework under more advanced sensing modalities. In addition, we will incorporate more comprehensive evaluation metrics, such as NDS, ATE, and ASE, to provide a more thorough and standardized assessment of detection performance. This will provide a more comprehensive understanding of the model’s strengths and limitations in practical applications.

## Figures and Tables

**Table 1:** Comparison of representative multimodal vehicle detection methods.

Method	Modality	Fusion Level	Fog Robust	Height Modeling	Sparsity Handling	Main Limitation
RadarNet [10]	R + L	Early + Late (Attention)	×	Approx.	Partial	Sparse radar limits accuracy
DEF [7]	R + L	Early	Partial	Approx.	×	Low radar resolution
MVDNet [8]	R + L	Attention-based	√	Approx.	Partial	Inaccurate height in fusion
Bi-LRFusion [42]	R + L	Bidirection-based	×	√	Partial	Incomplete height compensation for tall objects; residual sparsity
ST-MVDNet [9]	R + L	Multi-stage	√	Approx.	Partial	No explicit height modeling
TransFusion [11]	R + L	Two-stage	√	Partial	Partial	Complex temporal alignment
Tabassum et al. [12]	R + L	Multi-head Attention	√	Approx.	Partial	Still limited height accuracy
Timely Fusion [13]	R + L	Timely Fusion	√	Approx.	Partial	Dependency on synchronization

**Table 2:** Comparison of the experimental results of our proposed method with other state-of-the-art radar and LiDAR fusion methods on ORR dataset. “L” denotes “LiDAR”, while “L + R” signifies the combination of “LiDAR and Radar”.

Method	Train	Modality	Clear + Foggy (LiDAR)						Clear-Only					
	Test		Clear			Foggy (Lidar)			Clear			Foggy (Lidar)		
	IOU		0.5	0.65	0.8	0.5	0.65	0.8	0.5	0.65	0.8	0.5	0.65	0.8
PIXOR (CVPR2018) [34]		L	72.8	68.3	41.2	62.6	58.9	35.7	71.0	67.2	40.6	61.8	58.3	35.7
PointRCNN (CVPR2019) [26]			78.2	73.8	45.7	69.7	65.6	41.6	78.2	72.8	43.4	68.7	64.0	37.6
Pointpillars (CVPR2019) [29]			85.7	83.0	58.3	72.8	70.3	48.6	85.8	82.9	60.6	71.3	68.3	47.8
DEF (CVPR2020) [7]		L + R	86.6	78.2	46.2	81.4	72.5	41.1	85.9	78.1	44.2	71.8	63.7	32.4
MVDNet (CVPR2021) [8]			90.9	88.8	74.6	87.4	84.6	68.9	87.2	86.1	72.6	78.0	75.9	61.6
Timely Fusion (2024) [13]			89.8	86.9	72.2	-	-	-	-	-	-	-	-	-
Bi-LRFusion (CVPR2023) [42]			92.2	89.5	75.1	86.7	81.5	69.2	90.1	88.0	74.5	84.1	80.2	65.3
TransFusion (2024) [11]			91.8	89.6	75.4	88.6	85.3	70.1	88.2	87.2	74.0	79.1	76.3	63.2
Tabassum et al. (2024) [12]			91.2	88.9	74.1	-	-	-	-	-	-	-	-	-
ST-MVDNet (CVPR2022) [9]			94.7	93.5	80.7	91.8	88.3	73.6	91.4	89.9	78.4	81.2	80.8	64.9
MVDFusion			95.8	94.2	81.5	93.2	89.6	74.1	92.7	91.2	80.6	82.3	81.2	65.3

**Table 3:** The influence of individual modules on the performance of the proposed method is examined through experiments conducted on the ORR dataset.

Model	Train	DAM	RHQM	RLQFM	Clear + Foggy (LiDAR)						Clear-Only					
	Test				Clear			Foggy (LiDAR)			Clear			Foggy (LiDAR)		
	IOU				0.5	0.65	0.8	0.5	0.65	0.8	0.5	0.65	0.8	0.5	0.65	0.8
Model					87.3	79.6	60.3	83.2	74.8	57.6	85.6	78.4	58.7	72	66.4	50.8
		√			89.7	81.3	62.5	84.4	76.4	60	87.8	80.8	60.7	73.1	68.7	53.2
		√	√		93.3	85.7	66.4	90.8	80.8	67.9	91.7	86.4	69.1	77.1	73.5	60.4
		√		√	94.3	90.1	77.2	92.3	85.4	73.4	92	88.3	74.4	80.8	78.5	64.8
		√	√	√	95.8	94.2	81.5	93.2	89.6	74.1	92.7	91.2	80.6	82.3	81.2	65.3

## Data Availability

The Oxford Radar RobotCar dataset analyzed in this study is publicly and openly available from the Oxford Robotics Institute at https://oxford-robotics-institute.github.io/radar-robotcar-dataset/ (accessed on 3 April 2026) (also accessible via the main Oxford RobotCar Dataset portal at https://robotcar-dataset.robots.ox.ac.uk/) (accessed on 3 April 2026). No new data were created or generated during this study. Data sharing is not applicable beyond the publicly archived dataset referenced above.
